# Editorial: Towards a psychophysiological approach in physical activity, exercise, and sports, volume IV

**DOI:** 10.3389/fpsyg.2025.1648674

**Published:** 2025-07-08

**Authors:** Pedro Forte, José E. Teixeira, Daniel Portella, Diogo Monteiro

**Affiliations:** ^1^CI-ISCE, Higher Institute of Educational Sciences of the Douro, Penafiel, Portugal; ^2^Research Center for Active Living and Wellbeing (LiveWell), Polytechnic Institute of Bragança, Bragança, Portugal; ^3^Department of Sports Sciences, Instituto Politécnico de Bragança, Bragança, Portugal; ^4^Department of Sports Sciences, Polytechnic of Guarda, Guarda, Portugal; ^5^Department of Sports Sciences, Polytechnic of Cávado and Ave, Guimarães, Portugal; ^6^SPRINT—Sport Physical Activity and Health Research and Innovation Center, Guarda, Portugal; ^7^Master's Programme in Innovation in Higher Education in Health, Universidade Municipal de São Caetano do Sul, São Caetano do Sul, Brazil; ^8^ESECS, Instituto Politécnico de Leiria, Leiria, Portugal; ^9^Research Center in Sports, Health and Human Development, Covilhã, Portugal

**Keywords:** physical, activity, exercise, sports, psychophysiological

The Research Topic “*Toward a psychophysiological approach in physical activity, exercise, and sports, volume IV”* represents a significant stride forward in the integration of psychological and physiological paradigms in sport and exercise science. This editorial aims to synthesize insights from 23 contributions that deepen our understanding of the complex bidirectional interactions between mind and body in the context of physical activity, sports performance, exercise-based health, and wellbeing promotion.

A recurring focus across the Research Topic is the impact of physical activity on emotional regulation and wellbeing. Wang S. et al. demonstrated that Tai Chi significantly improved emotional regulation efficacy and subjective wellbeing in older adults, with emotion regulation acting as a partial mediator. Similarly, Jiang et al. identified that academic self-efficacy was enhanced through physical activity, mediated by future orientation and mental toughness.

Among youth populations, Zhang et al. found that parenting styles influenced sport adherence via goal orientation and self-regulation. Complementing this, Li and Zhou examined junior high school students, finding that self-esteem and interpersonal relationships jointly mediated the association between physical exercise and school adaptation. This research emphasizes the need for the multifaceted benefits of physical activity in developmental contexts.

From a high-performance perspective, Liu et al. showed that elite karate athletes had superior cognitive inhibition skills compared to sub-elite peers, supporting the neural efficiency hypothesis. Ilbak et al. further validated the relevance of psychophysiological monitoring by showing that perceived exertion aligned well with internal training load in combat sports.

On the public health front, a meta-analysis by Wang Y. et al. confirmed that replacing sedentary behavior with even light to moderate-to-vigorous physical activity significantly reduced the risk of depression. This was echoed by Li et al., who reported that consistent physical activity improved subjective wellbeing in university students, and this can be used as a valid strategy for the prevention of psychological disorders.

Innovative psychological skills training and emotion-focused interventions were prominent in this volume. Kequn et al. designed a mindfulness-based program that improved attentional focus, emotional regulation, and competitive performance. Yang et al. revealed that self-compassion decreased competitive anxiety, mediated by increases in confidence.

Adapted physical activity was also explored. Myraunet et al. identified intrinsic motivation as a key predictor of sports adherence among para-athletes, while Gao et al. showed that life skills such as autonomy and social competence were improved through physical activity in individuals with intellectual disabilities.

Dong and Lin demonstrated that social support—from peers and coaches—reduced anxiety and depression among adolescent team sport athletes. Ji et al. found that athletic identity was a significant predictor of post-retirement life satisfaction in former athletes.

Regarding performance optimization, Chatterjee et al. conducted a meta-analysis on neuromuscular warm-up protocols, reporting consistent benefits for strength, balance, and explosive power. Wang Z. et al. revealed that attentional control and emotional regulation were key predictors of performance consistency in basketball.

Liu et al. showed that physical self-efficacy mediated the link between confidence and accurate self-assessment of performance under physical stress, while Meixner et al. found that higher self-efficacy improved performance prediction accuracy under physically demanding conditions.

Wang D. et al. connected postural control with executive function in youth, revealing that postural stability predicts cognitive flexibility. In a separate study, Niu et al. reported that parental support positively influenced youth engagement in physical activity.

Drole et al. validated the Turkish version of the Sport Coping Skills Inventory-Revised, confirming its psychometric validity, reliability, and internal consistency. Additionally, the Enjoyment theme was also investigated in this Research Topic, a critical factor for adherence, which was the focus of Beck et al.'s work, comparing group vs. individual spinning sessions. Group settings were found to increase enjoyment, suggesting that social context enhances emotional experience during physical exercise. Finally, Jiang et al. examined gender differences in emotional benefits from exercise, concluding that women experience greater improvements in subjective wellbeing under similar workloads.

Taken together, these 23 articles reveal that physical activity, exercise, and sports are not just physiological experiences but fundamentally psychophysiological phenomena. Emotional regulation, confidence, motivation, and social relationships shape both outcomes and engagement to boost exercise engagement and maintain the habitual practice as well as health-related physical fitness, health, and performance.

However, limitations remain. There is a need for more longitudinal studies, better integration of physiological data (e.g., HRV, cortisol), and culturally diverse validation of psychological tools. Real-time, wearable-based monitoring and ecological assessment methods offer promising paths forward.

In conclusion, *Volume IV* provides a compelling case for embracing a biopsychosocial paradigm in sport and exercise science. These contributions affirm that human movement is as much a psychological and social act as it is a physical one, calling for interdisciplinary approaches that reflect the complexity of the active human being.

